# African American Adherence to COVID-19 Public Health Recommendations

**DOI:** 10.3928/24748307-20200707-01

**Published:** 2020-08-06

**Authors:** Ray Block, Arthur Berg, Robert P. Lennon, Erin L. Miller, Marcella Nunez-Smith

## Abstract

By mid-May 2020, most of the United States had been under shelter-in-place orders for several weeks to decrease the spread of coronavirus 2019 (COVID-19). As states begin to lift these orders to reopen the economy, the risk of a resurgence of COVID-19 may be related to the public's voluntary adherence to public health recommendations. We conducted a nationally representative survey of 604 African Americans to generate a risk assessment based on African Americans' compliance with public health recommendations to frequently wash hands, maintain social distancing, avoid touching face, and wear a mask in public. This is, to our knowledge, the most comprehensive study of African Americans and public health adherence specific to COVID-19. The percent of respondents reporting that they *always* comply with these recommendations was 72%, 67%, 55%, and 65%, respectively. Based on this threshold, African Americans' level of adherence with COVID-19 public health recommendations suggests they may be at high risk of a resurgence of COVID-19 during reopening, and there is an urgent need for targeted, culturally responsive public health messaging that is accessible to communities of color to help address racial disparities in COVID-19 risk. **[*HLRP: Health Literacy Research and Practice*. 2020;4(3):e166–e170.]**

Research models suggest that stay-at-home orders may mitigate the risk of community transmission of severe acute respiratory syndrome coronavirus 2 (SARS-CoV-2) and relieve the resulting stress on our health care systems (Nussbaumer-Streit et al., 2020). Yet, to minimize further economic damage (already estimated in the trillions of dollars in the United States alone (Centre for Risk Studies at the University of Cambridge Judge Business School, 2020), many states are moving to reopen their economies by lifting these shelter-in-place orders, (Vause, Baer, & Kiley, 2020) in spite of the risk of a resurgence in coronavirus 2019 (COVID-19) caused by reopening (Fox, 2020). This risk has already been demonstrated in Mississippi and other states (Porterfield, 2020). Balancing the risk of harm from COVID-19 resurgence against the risk of harm from pandemic-caused economic hardship is particularly challenging in African American communities, which are among the most negatively impacted economically by stay-at-home orders (Aratani & Rushe, 2020), and are also suffering some of the highest mortality rates from COVID-19 (Centers for Disease Control and Prevention, 2020). Adherence to public health recommendations will be critical to minimize resurgence in community transmission during reopening.

There are limited data on African American understanding of and compliance with public health recommendations pertaining to COVID-19. One study (*N* = 630) showed lower COVID-19 knowledge levels in ethnic/racial minority US populations but did not explicitly ask about compliance (Wolf et al., 2020). A larger study (*N* = 5,984) also showed lower knowledge in ethnic/racial US minority populations, and low intent to comply with Centers for Disease Control and Prevention recommendations, but had limited African American respondents (Van Scoy et al., 2020). We recently completed the first national poll that, to our knowledge, focuses exclusively on the African American experience with COVID-19. We sought to explore reported compliance with four key public health recommendations that are endorsed at federal and local levels to prevent future COVID-19 pandemic waves.

## Methods

The poll was conducted from May 1-7, 2020. The overall goal was to explore what African American people know and believe about COVID-19, how COVID-19 is affecting African Americans, and the perceived political implications of COVID-19. This article reports the intent to comply with public health recommendations. Details about the survey design, along with a slide deck discussing the items in the questionnaire, are available online at https://www.africanamericanresearch.us/survey-results. A data collection firm used weighted convenience sampling to generate a nationally representative sample of African Americans. To achieve a margin of error lower than 4%, 604 completed surveys were needed. The survey was developed by the African American Research Collaborative (AARC; www.africanamericanresearch.us), an organization committed to bringing an accurate understanding of African American civic engagement to the public discourse. Respondents were asked about these four public health recommendations: “frequently wash your hands for at least 20 seconds” (wash hands); “stay at least 6 feet away from others when in public” (social distancing); “avoid touching your eyes, nose, and mouth with un-washed hands” (avoid touching face); and “wear a mask when outside and could come in contact with others” (wear mask). Adherence with these recommendations was measured by asking participants to identify how often they follow those recommendations on a 4-point scale (*always*, *most of the time*, *not very often*, *never*). The National Association for the Advancement of Colored People and Equity Research and Innovation Center (ERIC) at Yale School of Medicine funded this public poll of African Americans by the AARC. Members of these funding organizations provided consultation on developing the poll. The Director of ERIC is a collaborator on this report, but otherwise the analyses happened independently of the expert consultants and funding agencies.

## Results

The study contacted a total of 1,399 African Americans, of whom 604 completed the survey for a completion rate of 43.2%. To ensure representation from those not online, 350 were contacted via telephone; the remaining 1,049 by email. Data were not collected on nonrespondents. The weighted demographic data (**Table 1**) are consistent with a national sample of African Americans, with a margin of error of ±3.9%. Weighted to represent a national sample, intent to *always* adhere to public health recommendations was wash hands (72%), social distancing (67%), avoid touching face (55%), and wear mask (65%) (**Table 2**).

## Discussion

The extent to which adherence with these recommendations determines the effectiveness of the recommendations is not known. One modeling study (pre-peer review) suggests that 90% compliance will still take more than 3 months to control COVID-19, 80% compliance might take more than 5 months to control the disease, and compliance lower than 70% will be ineffective at controlling the disease (Chang, Harding, Zachreson, Cliff, & Prokopenko, 2020). Using the *always* response categories on our poll items as a threshold for assessing intent, populations under 80% compliance, like African Americans, may expect worse health outcomes.

Although the reasons for low compliance are currently not known, a nuanced understanding of historical contexts and the lived experiences of African Americans is essential to interpreting data recorded about this marginalized group. For example, lower access to information about COVID-19 among ethnic/racial US minority populations (Van Scoy et al., 2020; Wolf et al., 2020) may partially explain lower compliance, as individual decision-making regarding behavior during a pandemic is driven by information, including awareness of disease rates at a local level, awareness of opportunities for testing and treatment, and understanding of how behavior may modify disease risk (Venkatesan, Nguyen-Van-Tam, & Siebers, 2019). This suggests that the racial disparities (Centers for Disease Control and Prevention, 2020) may stem in part not only from the ongoing effects of historic health inequity (Webb Hooper, Nápoles, & Pérez-Stable, 2020) but also from an ongoing inequality in access to COVID-19 information, which could be addressed immediately with targeted education campaigns, and may lead to increased compliance (Venkatesan et al., 2019) and decreased COVID-19 incidence and mortality.

## Study Limitations and Strengths

Our data are intentionally limited to African Americans and may not be generalizable to non-African Americans. Our poll is cross-sectional, representing a single moment, and may not be generalizable over time. This is particularly true given the social unrest triggered by the police killings of George Floyd and many other unarmed African Americans, which challenges social distancing recommendations, increasing health risks among protesters (Stracqualursi, 2020). Given the nationwide protests, African American compliance with social distancing is likely lower now. This is concerning, as interventions like social distancing are estimated to have prevented 60 million COVID-19 infections in the US (Hsiang et al., 2020). However, pandemic response is a struggle for hegemony between science and politics (Hall & Wolf, 2019). Science may describe the pathogen and mitigation strategies, and develop treatments, vaccines, and cures; politics, however, determines the response and further informs individual behavior.

The forgoing of public health recommendations in response to the current social unrest should be considered in that light, and it is an important element in unlocking the value of our results. The purpose of quantifying compliance with public health recommendations is to estimate risk from resurgence and to inform mitigation strategies. As the political landscape changes, dynamic interpretation of prior data is warranted; understanding motivations for lower compliance is imperative to developing mitigation strategies. Some have criticized protesters for not following COVID-19 public health recommendations (Flood, 2020). This criticism may be an appropriate admonishment for protesters not wearing masks. However, there is little evidence of the protests leading to spikes in COVID-19 cases (Dave, Friedson, Matsuzawa, Sabia & Safford, 2020), and research suggests that premature openings in some states pose a greater risk to African Americans (Brueck, 2020; Rapfogel & Calsyn, 2020). Perhaps more importantly, the conscious decision to eschew social distancing recommendations to join protests against racial inequality is entirely rational for protesters who have determined that their increased risk of COVID-19 from marching is a necessary risk to combat racial injustice.

Our study has several strengths. It is the only targeted African American poll to assess compliance with COVID-19–related public health recommendations. By using best-practice polling methods and weighted sampling, our sample is generalizable to African Americans within a margin of error of ± 3.9%. This gives African American communities a measure of their relative risk, which will inform their balance of economic need versus health risk (Abrams & Block, 2020). This may also inspire elected officials to target compliance efforts in African American communities, African American community leaders to emphasize compliance with their constituents, and the media to help shape and support a public narrative that increases compliance. Combined, these efforts may to help improve the rates of COVID-19 exposure in African Americans.

In summary, this study reports that African Americans have lower than 80% adherence to four key COVID-19 public health recommendations. Based on this threshold, targeted education campaigns and consistent messaging across political groups, African American community leaders, and media outlets to improve compliance, in turn decreasing risk, are indicated. Ongoing social upheaval may dramatically change behavior and increase risk. Strategies to adapt mitigation techniques to dynamic social conditions are indicated. Given the substantial racial inequality of COVID-19 outcomes, the COVID-19 pandemic is a fitting backdrop to protests for racial equality, and a reminder of a perhaps lesser-known observation of Dr. Martin Luther King, Jr.: “Of all the forms of inequality, injustice in health is the most shocking and the most inhuman because it often results in physical death” (King, 1966).

## Figures and Tables

**Table 1:** Weighted Respondent Demographics (*N* = 604)

**Characteristic**	**%**
Gender	
Female	53.3
Male	45.9
Nonbinary	0.8

Education	
Less than high school	5.7
High school graduate or GED	34.1
Some college, but did not graduate	21.5
2-year degree, trade, or technical school	10.4
4-year degree/Bachelor's degree	17.6
Graduate or professional degree	9.8
Prefer not to say	0.7

Region	
South	51.4
Midwest	20.2
Northeast	18.2
West	10.2

Note. Margin of error ±3.9%. GED = General Educational Development.

**Table 2:** Compliance with COVID-19 Public Health Recommendations Weighted to National Representation (*N* = 604)

**How often do you follow these recommendations regarding protecting yourself and others from COVID-19?**	**Always, %**	**Most of the time, %**	**Not very often, %**	**Never, %**
Frequently wash your hands for at least 20 seconds	72	21	4	4
Stay at least 6 feet away from others when in public	67	23	5	4
Avoid touching your eyes, nose, and mouth with unwashed hands	55	31	10	4
Wear a mask when outside and could come into contact with others	65	18	8	9

Note. Margin of error ±3.9%. COVID-19 = coronavirus 2019.
